# Native T1 mapping in ATTR cardiac amyloidosis - comparison with AL cardiac amyloidosis - a 200 patient study

**DOI:** 10.1186/1532-429X-16-S1-O4

**Published:** 2014-01-16

**Authors:** Marianna Fontana, Sanjay M Banypersad, Thomas A Treibel, Viviana Maestrini, Daniel Sado, Steven K White, Silvia Castelletti, Anna S Herrey, Philip N Hawkins, James Moon

**Affiliations:** 1 Heart Hospital Imaging Center, Heart Hospital, University College London, London, UK; 2 National Amyloidosis Center, Royal Free Hospital, University College London, London, UK

## Background

Transthyretin amyloidosis (ATTR amyloidosis) is an under diagnosed cause of heart failure with no truly quantitative test. Cardiac involvement is the leading cause of death and influences therapeutic choices. Since new therapies are imminent which aim to treat ATTR amyloidosis the lack of a quantitative test represents a critical step for drug development. In cardiac AL amyloidosis, T1 has high diagnostic accuracy and tracks disease. We hypothesised similar results would occur for ATTR. We hypothesized that the native myocardial T1 would be elevated in ATTR amyloid; that T1 elevation would track the cardiac amyloid burden as measured by DPD grading; and that T1 elevation would be an early disease marker.

## Methods

3 groups were studied: ATTR amyloid patients (n = 85; 70 male; age 73 ± 10); healthy mutations carriers (n = 8; 3 male; age 47 ± 6); and AL amyloid patients (n = 79; 55 male; age 62 ± 10). These were compared with 52 healthy volunteers and 46 patients with hypertrophic cardiomyopathy (HCM). All underwent T1 mapping (Shortened Modified Look-Locker Inversion recovery, ShMOLLI). ATTR patients and mutation carriers also underwent cardiac DPD scintigraphy.

## Results

**Figure 1:**
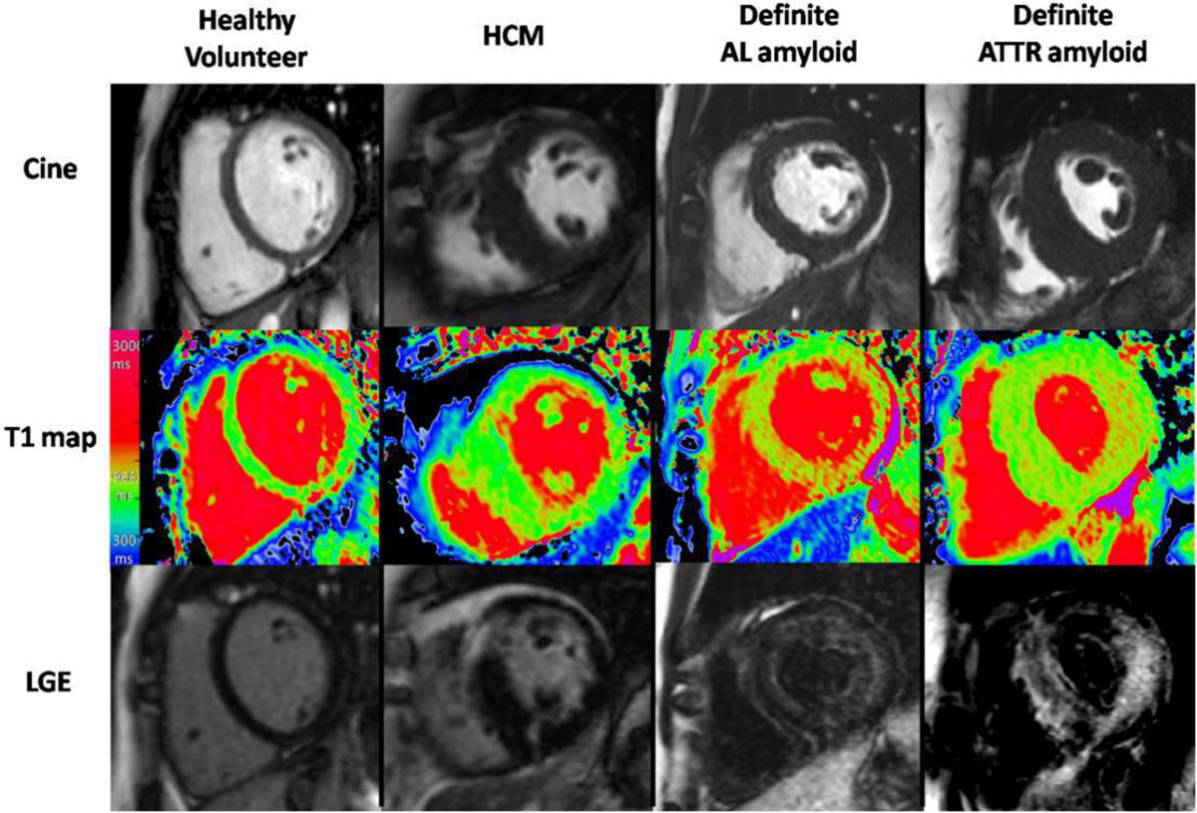
**Characteristic examples from CMR scans - CMR end-diastolic cine still (upper panel); ShMOLLI native T1 map (middle) and late gadolinium enhancement (LGE) images (lower) in (left to right) healthy volunteer, HCM, definite AL and definite ATTR patients**. Note the markedly elevated myocardial T1 time in the AL cardiac amyloid patient and ATTR patient into the red range of the colour scale (the elevation is higher in AL, i.e. more red) compared to the normal control (green) and the patient with hypertrophic cardiomyopathy.

**Figure 2:**
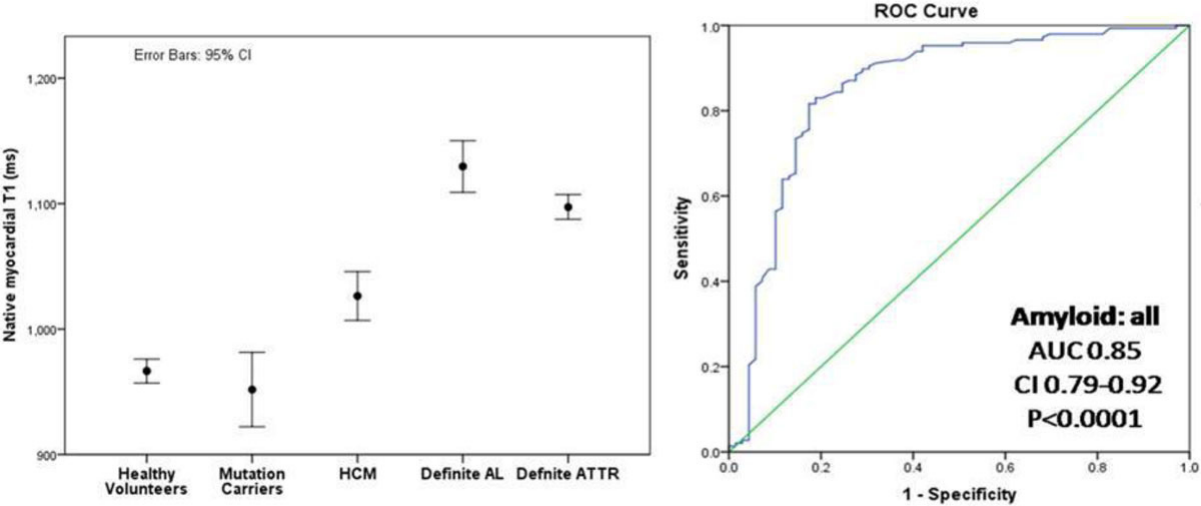
**ROC curve for native T1 - Left pane: Native T1 in healthy volunteers, mutation carriers, HCM, definite AL and definite ATTR**. Right panel: Receiver-operating characteristic (ROC) curve for the discrimination of possible or definite cardiac amyloid by native myocardial T1 from HCM.

T1 was elevated in ATTR patients compared to HCM and normal subjects (1097 ± 43 ms vs 1026 ± 64 ms vs 967 ± 34 ms, both p < 0.0001). In established cardiac ATTR amyloidosis, T1 elevation was not as high as in AL amyloidosis (AL 1130 ± 68 ms, p = 0.01) (Figure 1 and 2). Diagnostic performance was similar for AL and ATTR amyloid (vs HCM: AL AUC 0.84 (95%CI 0.76-0.92); ATTR 0.85 (0.77-0.92) P < 0.0001) (Figure 2). T1 correlated with cardiac amyloid burden as determined semi-quantitatively by DPD scintigraphy (p < 0.0001). T1 was not elevated in mutation carriers (952 ± 35 ms) but was in isolated DPD grade 1 (n = 9, 1037 ± 60 ms, p = 0.001).

## Conclusions

Native myocardial T1 mapping represents the first test able in ATTR amyloidosis to quantify the cardiac amyloid burden. Native myocardial T1 detects cardiac ATTR amyloid with similar diagnostic performance and potential for quantitation as AL amyloid, but with lower maximal T1 elevation and appears to be an early marker of disease.

## Funding

Dr Fontana Is funded by the British Heart Foundation. A proportion of the scans have been funded by GlaxoSmithKline.

